# Association between placental histological chorioamnionitis and respiratory distress syndrome in preterm infants: a retrospective cohort study

**DOI:** 10.3389/fped.2026.1910761

**Published:** 2026-08-27

**Authors:** Hua Ke, Dong Yang, Liangjuan Zhang, Chengyin Liu

**Affiliations:** Neonatology Department, Northwest Women’s and Children’s Hospital, Xi’an, Shaanxi, China

**Keywords:** gestational age, histological chorioamnionitis, placental pathology, preterm infants, respiratory distress syndrome, risk stratification

## Abstract

**Objective:**

To evaluate the independent association between placental histological chorioamnionitis (HCA) and respiratory distress syndrome (RDS) in preterm infants and explore whether this association varied by gestational age and histological stage. The incremental predictive value of HCA beyond gestational age and birth weight was also assessed.

**Methods:**

This single-center retrospective cohort study included 689 preterm infants born at <34 weeks of gestation between June 2020 and June 2025 with definitive placental histopathological findings; 392 were HCA-negative and 297 were HCA-positive. Multivariable logistic regression assessed the association between HCA and RDS after adjustment for gestational age, birth weight, premature rupture of membranes, antenatal corticosteroid exposure, and maternal C-reactive protein. An HCA × gestational-age-group interaction was tested. Three exploratory models were compared: HCA alone, gestational age plus birth weight, and gestational age plus birth weight plus HCA. Model performance was evaluated using AUC, DeLong and likelihood-ratio tests, reclassification indices, calibration, and 1,000 bootstrap resamples.

**Results:**

Overall, 271 infants (39.33%) developed RDS. RDS incidence was lower in the HCA-positive group than in the HCA-negative group (31.99% vs. 44.90%, *P* < 0.001). The incidences of RDS in stage I, II, and III HCA were 26.32%, 30.61%, and 55.56%, respectively (*P* = 0.004). Among infants born at <32 weeks, RDS incidence was lower in the HCA-positive group (33.81% vs. 52.94%, *P* = 0.001), whereas no significant difference was observed at 32–<34 weeks (30.38% vs. 38.74%, *P* = 0.093). After adjustment, HCA remained associated with lower odds of RDS (adjusted OR = 0.654, 95% CI: 0.472–0.906, *P* = 0.011), but the interaction with gestational-age group was not significant (*P* = 0.184). HCA alone showed poor discrimination (AUC=0.566). Adding HCA to gestational age and birth weight increased the AUC from 0.824 to 0.829, but the difference was not significant (*P* = 0.218). Bootstrap-corrected AUCs were 0.821 and 0.825.

**Conclusion:**

Placental HCA was independently associated with lower odds of RDS, but this observational association does not establish a causal protective effect. Gestational-age- and stage-specific findings were exploratory. HCA alone had poor discrimination, and its incremental predictive contribution beyond gestational age and birth weight was modest.

## 1 Introduction

Respiratory distress syndrome (RDS) is a major cause of early respiratory failure and adverse outcomes in preterm infants. Its pathogenesis is primarily related to structural lung immaturity and insufficient pulmonary surfactant production. Consequently, the risk of RDS increases substantially with decreasing gestational age and birth weight (1). Although advances in antenatal corticosteroid administration, surfactant replacement, and noninvasive respiratory support have improved neonatal outcomes, RDS remains an important clinical problem, particularly among very and extremely preterm infants. Identification of perinatal factors associated with RDS may improve understanding of disease heterogeneity and facilitate individualized postnatal assessment. Histological chorioamnionitis (HCA) is a placental manifestation of intrauterine inflammation characterized by neutrophilic infiltration of the placental membranes, chorionic plate, or related tissues. HCA is frequently identified in preterm deliveries and may influence fetal lung development through inflammatory mediators and alterations in pulmonary maturation (2–4). Experimental and clinical evidence suggests that limited prenatal inflammatory exposure may accelerate maturation of type II alveolar epithelial cells and increase surfactant protein expression, potentially reducing the occurrence of classical surfactant-deficiency RDS. Conversely, severe or prolonged inflammation, particularly when accompanied by fetal inflammatory response or funisitis, may contribute to pulmonary injury, impaired alveolar development, and subsequent bronchopulmonary dysplasia (5–7). Therefore, intrauterine inflammation may exert different respiratory effects according to its severity, duration, anatomical extent, and involvement of the fetal compartment.

Previous observational studies and systematic reviews have evaluated the association between chorioamnionitis and neonatal respiratory outcomes; however, their findings regarding RDS remain inconsistent. Some studies have reported a lower incidence of RDS among infants exposed to HCA, whereas others have found no independent association after adjustment for gestational age and other perinatal characteristics. These discrepancies may be partly attributable to differences in study populations, definitions of chorioamnionitis, gestational-age distributions, antenatal management, and adjustment for major developmental factors. In addition, many previous studies have classified HCA only as present or absent, without considering the histological stage of the maternal inflammatory response. Evidence is also limited regarding whether the association between HCA and RDS differs between infants born before 32 weeks and those born at 32 to less than 34 weeks of gestation. Moreover, because placental pathological results are generally unavailable during initial neonatal respiratory management, the clinical relevance of HCA should primarily be considered in terms of etiological interpretation and supplementary postnatal risk assessment rather than real-time prediction. Therefore, the primary objective of the present study was to evaluate the independent association between placental HCA and RDS in preterm infants born at less than 34 weeks of gestation after adjustment for major maternal and perinatal confounders. Secondary objectives were to explore whether this association varied according to gestational-age strata and the histological stage of the maternal inflammatory response. The discriminatory performance of HCA alone and of models integrating HCA with gestational age and birth weight was evaluated as an exploratory analysis rather than for the development of a definitive clinical prediction model.

## 2 Materials and methods

### 2.1 General information

This single-center retrospective cohort study included preterm infants born at <34 weeks of gestation who were admitted to the Neonatal Intensive Care Unit of our hospital between June 2020 and June 2025, together with their corresponding maternal clinical data. Placental histopathological examination was performed selectively according to additional clinical indications, including premature rupture of membranes, maternal fever, suspected intrauterine infection, abnormal amniotic fluid, and other high-risk perinatal conditions. Preterm birth alone did not necessarily result in placental pathological examination; therefore, placental histopathology was not routinely available for all deliveries before 34 weeks of gestation. Only infants with available and definitive placental histopathological findings were included. Infants born to mothers with clinical chorioamnionitis were excluded because this condition represents a clinically apparent intrauterine infectious-inflammatory process that is frequently accompanied by maternal systemic manifestations, urgent obstetric intervention, and peripartum antibiotic treatment. These clinical features and treatment-related factors may independently influence neonatal respiratory outcomes and introduce substantial clinical heterogeneity. Accordingly, the present study primarily focused on the association between subclinical histological placental inflammation and neonatal respiratory distress syndrome.

#### 2.1.1 Inclusion criteria

1. Preterm infants with gestational age <34 weeks;
2. Singleton pregnancy;
3. Admission to the NICU of our hospital after birth;
4. Placental examination performed by routine histopathology with a definitive diagnosis regarding the presence or absence of HCA;
5. Complete clinical data available, including perinatal maternal information and major neonatal outcome indicators.

#### 2.1.2 Exclusion criteria

1. Clinical chorioamnionitis;
2. Severe congenital malformations or chromosomal abnormalities;
3. Inherited metabolic diseases;
4. Missing perinatal clinical data or placental pathological data;
5. Death within 24 h after birth preventing assessment of RDS diagnosis.

**Figure 1:**
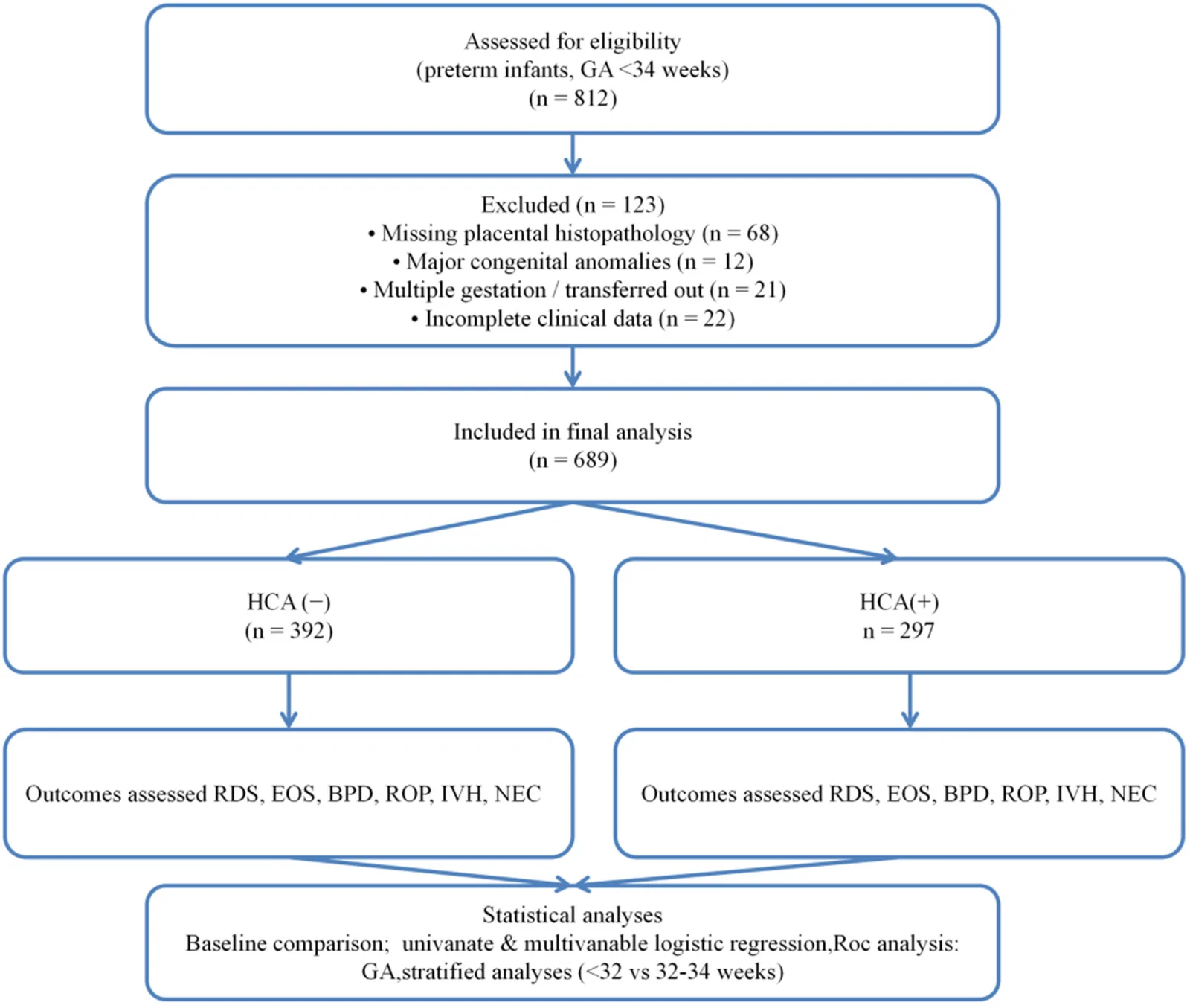
Study flowchart.

The study flowchart is shown in Figure 1.

### 2.2 Grouping

**Figure 2:**
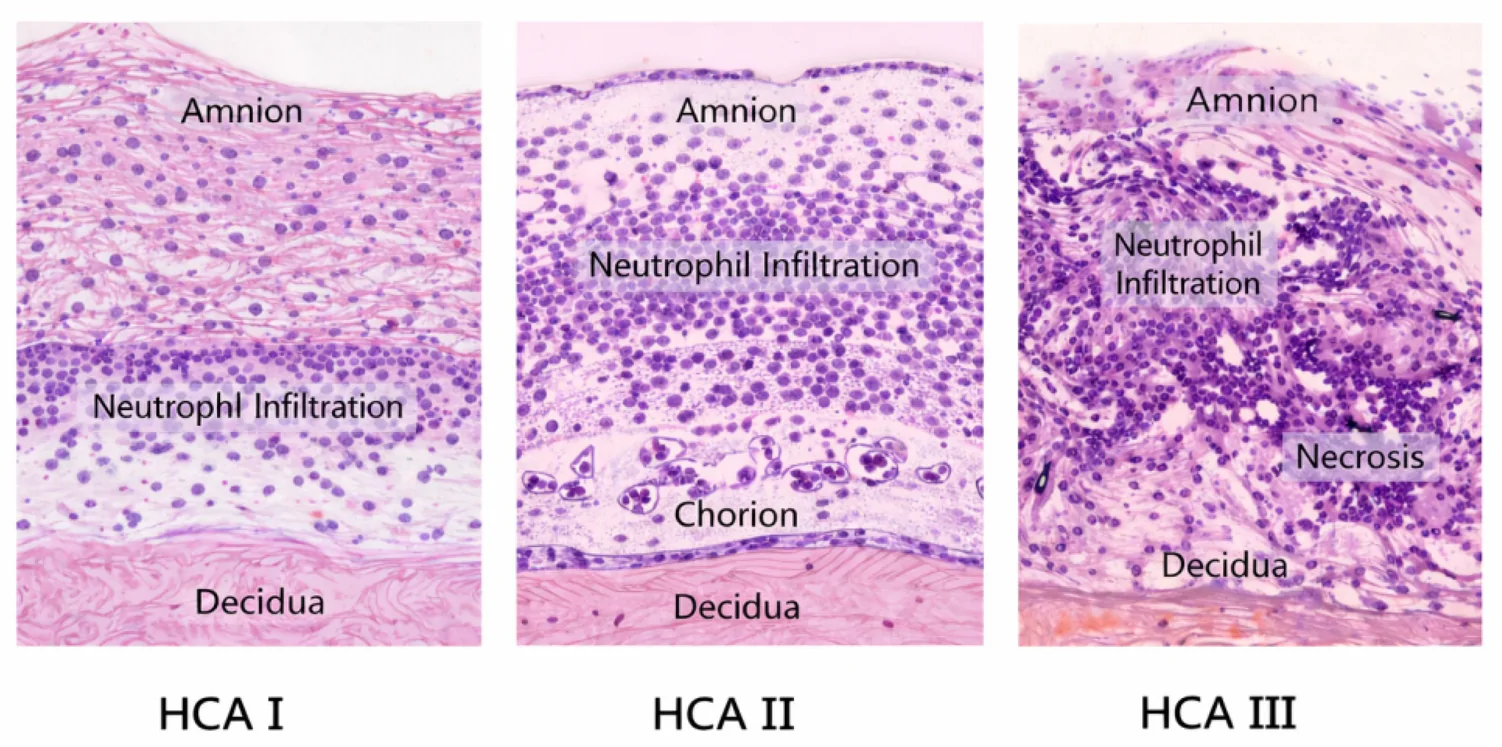
Diagnostic criteria for HCA staging.

According to placental histopathological findings, the enrolled preterm infants were classified into an HCA-negative group and an HCA-positive group. The diagnosis and histological staging of HCA were performed by experienced pathologists in accordance with the Amsterdam Placental Workshop Group Consensus Statement (8) (see Figure 2). HCA was defined by neutrophilic infiltration of the chorion, amnion, subchorionic space, or chorionic plate, reflecting a maternal inflammatory response. The maternal inflammatory response was classified into three stages: stage I, acute subchorionitis or chorionitis, characterized by neutrophilic infiltration of the subchorionic region or chorionic tissues; stage II, acute chorioamnionitis, characterized by extension of neutrophilic infiltration into the chorionic connective tissue and/or amnion; and stage III, necrotizing chorioamnionitis, characterized by prolonged inflammation with amniotic epithelial necrosis, basement membrane thickening, or more extensive tissue injury. The histological stages evaluated in the present study represented the maternal inflammatory response. Fetal inflammatory response, including funisitis and chorionic plate vasculitis, was not systematically recorded in the original pathological reports and therefore could not be classified or analyzed separately in this retrospective study. For exploratory subgroup analysis, the study population was stratified by gestational age into the <32-week group and the 32–<34-week group. According to neonatal respiratory outcomes, the infants were also categorized into the RDS group and the non-RDS group.

### 2.3 Data collection

In this study, perinatal clinical data of eligible preterm infants and their mothers were retrospectively collected from the hospital's electronic medical record system. Maternal data included age, gravidity, parity, gestational diabetes mellitus (GDM), hypertensive disorders of pregnancy, premature rupture of membranes (PROM) and its duration, presence of prenatal infection, use of antenatal antibiotics, administration of antenatal corticosteroids, and mode of delivery. Neonatal data included gestational age (GA), birth weight (BW), sex, Apgar scores at 1 and 5 min, need for resuscitation at birth, and requirement for respiratory support, Small for gestational age (SGA) was defined as a birth weight below the 10th percentile for gestational age and sex according to the Fenton preterm growth chart. Infants with a birth weight at or above the 10th percentile were classified as non-SGA (9). Perinatal inflammatory indicators included maternal white blood cell (WBC) count and C-reactive protein (CRP) levels. Early neonatal laboratory results (within 24 h after birth) included the first measured white blood cell count (WBC), absolute neutrophil count, lymphocyte proportion, hemoglobin level, platelet count, C-reactive protein (CRP), procalcitonin (PCT), interleukin-6 (IL-6), interleukin-8 (IL-8), blood pH value, arterial partial pressure of oxygen (PaO₂), partial pressure of carbon dioxide (PaCO₂), and lactate level. The incidences of bronchopulmonary dysplasia (BPD), early-onset sepsis (EOS), intraventricular hemorrhage (IVH), and necrotizing enterocolitis (NEC) were also recorded. BPD was defined as the requirement for supplemental oxygen at a concentration greater than 21% for at least 28 cumulative days. BPD status was assessed at 36 weeks’ postmenstrual age or discharge for infants born at <32 weeks of gestation and at 56 days of postnatal age or discharge for infants born at ≥32 weeks of gestation, whichever occurred first (10). All data were independently extracted and cross-checked by two investigators to ensure accuracy and completeness. Fetal inflammatory response, including funisitis and chorionic plate vasculitis, was not systematically recorded in the original pathological reports and therefore could not be analyzed separately in the present retrospective study. RDS was diagnosed according to established neonatal clinical criteria and the European Consensus Guidelines on the Management of Respiratory Distress Syndrome (1). The diagnosis was based on the presence of respiratory distress shortly after birth, including tachypnea, grunting, nasal flaring, chest retractions, and/or cyanosis, together with a requirement for supplemental oxygen or respiratory support and compatible chest radiographic findings. Alternative causes of neonatal respiratory distress, including transient tachypnea of the newborn, pneumonia, congenital heart disease, pulmonary hemorrhage, and air-leak syndromes, were excluded.

### 2.4 Statistical analysis

Statistical analyses were performed using SPSS version 26.0 and R software version 4.3.2. Continuous variables were assessed for normality using the Shapiro–Wilk test and are presented as mean ± standard deviation or median (P25, P75), as appropriate. Between-group comparisons were performed using the independent-samples t-test or Mann–Whitney U test. Categorical variables are presented as *n* (%) and were compared using the *χ*^2^ test or Fisher's exact test. Univariate logistic regression was used to identify factors associated with RDS. Variables with *P* < 0.10 and clinically relevant covariates were considered for multivariable analysis. The primary model included HCA, gestational age, birth weight, premature rupture of membranes, antenatal corticosteroid exposure, and maternal C-reactive protein. Neonatal white blood cell count was excluded from the primary model because it was measured after birth and might represent an intermediate variable but was added in a sensitivity analysis. SGA was not entered simultaneously with gestational age and birth weight because its definition incorporates both variables. Multicollinearity was evaluated using tolerance and variance inflation factors, with tolerance >0.20 and VIF <5 indicating no substantial multicollinearity. Effect modification by gestational age was assessed by adding an HCA × gestational-age-group interaction term to the model. Three exploratory prediction models were evaluated: Model 1 included HCA alone, Model 2 included gestational age and birth weight, and Model 3 additionally included HCA. Discrimination was assessed using the AUC with 95% confidence intervals, and Models 2 and 3 were compared using the DeLong test. The incremental value of HCA was evaluated using the likelihood-ratio test, continuous net reclassification improvement, and integrated discrimination improvement. Calibration was assessed using calibration plots, calibration intercepts, calibration slopes, and Brier scores. Internal validation was performed using 1,000 bootstrap resamples. Cases with missing placental pathology, RDS outcome, or key covariate data were excluded during cohort selection. No data were missing for variables included in the final models; therefore, complete-case analysis was performed without imputation. All tests were two-sided, and *P* < 0.05 was considered statistically significant.

## 3 Results

### 3.1 General characteristics

A total of 689 preterm infants were included, comprising 392 infants in the HCA-negative group and 297 infants in the HCA-positive group. Compared with the HCA-negative group, the HCA-positive group had lower gestational age and birth weight; higher proportions of premature rupture of membranes, maternal fever before delivery, positive vaginal secretion smear, and antenatal antibiotic exposure; a lower incidence of RDS; higher incidences of early-onset sepsis and neonatal leukemoid reaction; higher white blood cell and absolute neutrophil counts; and lower lymphocyte and platelet counts and hemoglobin levels at 2 h after birth (all *P* < 0.05). No significant between-group differences were observed in sex, 1- and 5-min Apgar scores, SGA status, maternal age, cesarean delivery, hypertensive disorders of pregnancy, gestational diabetes mellitus, antenatal corticosteroid exposure, antenatal magnesium sulfate exposure, bronchopulmonary dysplasia, retinopathy of prematurity, intraventricular hemorrhage, periventricular leukomalacia, necrotizing enterocolitis, or red blood cell count (all *P* > 0.05), as shown in Table 1.

**Table 1:** Comparison of baseline characteristics between the HCA-positive and HCA-negative groups.

Variable	HCA (-) (*n* = 392)	HCA (+) (*n* = 297)	t/*χ*^2^/z	*P*
Neonatal characteristics				
Gestational age (weeks, $\bar{x}$±s)	32.02 ± 1.59	31.44 ± 1.82	4.620	<0.001
Birth weight (g, $\bar{x}$±s)	1649.38 ± 294.62	1519.74 ± 309.53	5.737	<0.001
SGA, *n* (%)	48 (12.24)	31 (10.44)	0.544	0.461
Sex, *n* (%)			0.111	0.739
Male	214 (54.59)	166 (55.89)		
Female	178 (45.41)	131 (44.11)		
1-min Apgar score [*M* (*P*_25_, *P*_75_)]	7 (6, 8)	7 (6, 8)	−0.648	0.517
5-min Apgar score, M (P25, P75)	9 (8, 9)	8 (8, 9)	−0.967	0.334
Maternal and perinatal factors				
Maternal age, years	29.84 ± 4.61	30.22 ± 4.78	−1.087	0.277
Cesarean delivery, *n* (%)	248 (63.27)	179 (60.27)	0.648	0.421
Hypertensive disorders of pregnancy, *n* (%)	42 (10.71)	24 (8.08)	1.353	0.245
Gestational diabetes mellitus, *n* (%)	38 (9.69)	24 (8.08)	0.537	0.464
Premature rupture of membranes, *n* (%)	124 (31.63)	157 (52.86)	31.486	<0.001
Maternal fever before delivery, *n* (%)	21 (5.36)	48 (16.16)	19.732	<0.001
Positive vaginal secretion smear, *n* (%)	18 (4.59)	41 (13.81)	17.286	<0.001
Antenatal corticosteroid exposure, *n* (%)	286 (72.96)	221 (74.41)	0.170	0.680
Antenatal magnesium sulfate exposure, *n* (%)	102 (26.02)	85 (28.62)	0.603	0.437
Antenatal antibiotic exposure, *n* (%)	89 (22.70)	134 (45.12)	40.521	<0.001
Neonatal outcomes				
Respiratory distress syndrome, *n* (%)	176 (44.90)	95 (31.99)	11.805	<0.001
Early-onset sepsis, *n* (%)	47 (11.99)	72 (24.24)	17.892	<0.001
Bronchopulmonary dysplasia, *n* (%)	64 (16.33)	38 (12.80)	1.535	0.215
Retinopathy of prematurity, *n* (%)	49 (12.50)	41 (13.81)	0.255	0.613
Intraventricular hemorrhage, *n* (%)	58 (14.80)	53 (17.85)	1.102	0.294
Periventricular leukomalacia, *n* (%)	18 (4.59)	20 (6.73)	1.500	0.221
Necrotizing enterocolitis, *n* (%)	23 (5.87)	27 (9.09)	2.804	0.094
Neonatal leukemoid reaction, *n* (%)	14 (3.57)	33 (11.11)	14.733	<0.001
Laboratory parameters at 2 h after birth				
WBC (×10⁹/L, $\bar{x}$±s）	13.13 ± 4.18	15.61 ± 5.09	−6.963	<0.001
NEUT (×10⁹/L, $\bar{x}$±s）	8.21 ± 3.10	10.98 ± 3.87	−9.481	<0.001
LYMPH (×10⁹/L, $\bar{x}$±s）	3.94 ± 1.28	3.13 ± 1.17	8.765	<0.001
PLT (×10⁹/L, $\bar{x}$±s）	226.49 ± 62.32	210.39 ± 58.74	3.472	0.001
RBC (×10^12^/L, $\bar{x}$±s)	4.13 ± 0.59	4.07 ± 0.60	1.164	0.245
HGB (g/L, $\bar{x}$±s)	153.28 ± 18.73	149.52 ± 19.42	2.487	0.013

### 3.2 Comparison of RDS incidence according to HCA status and gestational age stratification

A total of 689 preterm infants were included in this study, among whom the overall incidence of RDS was 39.33% (271/689). The incidence of RDS also differed significantly across the three HCA stages (*χ^2^* = 11.005, *P* = 0.004), with rates of 26.32%, 30.61%, and 55.56% in stage I, stage II, and stage III HCA, respectively, indicating a relatively higher incidence among infants with stage III HCA. Gestational age-stratified analysis showed that, among infants born at <32 weeks of gestation, the incidence of RDS was significantly lower in the HCA-positive group than in the HCA-negative group (33.81% vs. 52.94%; *χ^2^* = 11.338, *P* = 0.001). Among infants born at 32–<34 weeks of gestation, the incidence of RDS was also lower in the HCA-positive group than in the HCA-negative group (30.38% vs. 38.74%), although the difference was not statistically significant (*χ^2^* = 2.825, *P* = 0.093) (see Table 2).

**Table 2:** Comparison of RDS incidence according to HCA status and gestational age stratification.

Group	Total (*n*)	RDS (*n*)	Incidence (%)	*χ^2^*	*P*
Overall comparison				11.805	<0.001
HCA (-)	392	176	44.90		
HCA (+)	297	95	31.99		
HCA stage				11.005	0.004
Stage I	114	30	26.32		
Stage II	147	45	30.61		
Stage III	36	20	55.56		
Gestational age <32 weeks				11.338	0.001
HCA (-)	170	90	52.94		
HCA (+)	139	47	33.81		
Gestational age 32–＜34 weeks				2.825	0.093
HCA (-)	222	86	38.74		
HCA (+)	158	48	30.38		

### 3.3 Association between HCA and major neonatal outcomes after adjustment for confounding factors

Separate logistic regression models were constructed to evaluate the associations between HCA and major neonatal outcomes. The coding and assignment of the variables included in the regression analyses are presented in Table 3. In the univariate analyses, HCA was associated with lower odds of RDS (OR = 0.577, 95% CI: 0.421–0.791, *P* < 0.001) and higher odds of early-onset sepsis (OR = 2.349, 95% CI: 1.568–3.519, *P* < 0.001). No statistically significant associations were observed between HCA and bronchopulmonary dysplasia, retinopathy of prematurity, intraventricular hemorrhage, or necrotizing enterocolitis (all *P* > 0.05), as shown in Table 4. After adjustment for potential confounding factors, HCA remained independently associated with lower odds of RDS (adjusted OR = 0.654, 95% CI: 0.472–0.906, *P* = 0.011). However, the adjusted associations between HCA and early-onset sepsis, bronchopulmonary dysplasia, retinopathy of prematurity, intraventricular hemorrhage, and necrotizing enterocolitis were not statistically significant (all *P* > 0.05), as shown in Table 5.

**Table 3:** Variable assignment.

Variable	Coding
RDS	0 = No, 1 = Yes
EOS	0 = No, 1 = Yes
BPD	0 = No, 1 = Yes
ROP	0 = No, 1 = Yes
IVH	0 = No, 1 = Yes
NEC	0 = No, 1 = Yes
HCA	0 = Negative, 1 = Positive
HCA stage	0 = Negative, 1 = Stage I, 2 = Stage II, 3 = Stage III
Gestational age	Continuous (weeks)
Birth weight	Continuous (g)
Sex	0 = Female, 1 = Male
PROM	0 = No, 1 = Yes
Delivery mode	0 = Vaginal, 1 = Cesarean
Antenatal corticosteroids	0 = No, 1 = Yes
Antenatal antibiotics	0 = No, 1 = Yes
Maternal CRP	Continuous
Neonatal WBC	Continuous
SGA	0 = No, 1 = Yes

**Table 4:** Univariate logistic regression analysis of the association between HCA and major neonatal outcomes.

Variable	β	SE	Wald *χ*^2^	*P* value	OR	95% CI
RDS	−0.549	0.161	11.657	<0.001	0.577	0.421∼0.791
EOS	0.854	0.205	17.352	<0.001	2.349	1.568∼3.519
BPD	−0.285	0.221	1.664	0.197	0.752	0.488∼1.160
ROP	0.114	0.229	0.248	0.615	1.121	0.718∼1.750
IVH	0.224	0.212	1.117	0.282	1.251	0.832∼1.880
NEC	0.473	0.293	2.597	0.109	1.604	0.900∼2.859

**Table 5:** Multivariate logistic regression analysis of the association between HCA and major neonatal outcomes.

Variable	β	SE	Wald *χ*^2^	*P* value	OR	95% CI
RDS	−0.425	0.168	6.400	0.011	0.654	0.472∼0.906
EOS	0.211	0.219	0.927	0.336	1.235	0.804∼1.896
BPD	−0.118	0.186	0.402	0.526	0.889	0.616∼1.284
ROP	0.094	0.191	0.242	0.623	1.099	0.754∼1.602
IVH	0.135	0.183	0.545	0.460	1.144	0.800∼1.637
NEC	0.211	0.231	0.835	0.361	1.235	0.785∼1.942

Each outcome was analyzed using a separate multivariable logistic regression model. All models were adjusted for gestational age, birth weight, premature rupture of membranes, antenatal corticosteroid exposure, and maternal CRP.

### 3.4 Univariate and multivariable logistic regression analyses for RDS

Univariate logistic regression analysis showed that HCA, gestational age, birth weight, 1- and 5-min Apgar scores, maternal fever before delivery, and antenatal corticosteroid exposure were associated with RDS (all *P* < 0.05). Platelet count met the prespecified screening criterion of *P* < 0.10 but was not retained in the primary model because of limited clinical relevance and the possibility that it reflected a postnatal biological response. SGA status was not significantly associated with RDS (OR = 1.189, 95% CI: 0.740–1.911, *P* = 0.474). The complete univariate analysis results are presented in Table 6. Based on the univariate results and clinical relevance, HCA status, gestational age, birth weight, premature rupture of membranes, antenatal corticosteroid exposure, and maternal C-reactive protein level were selected for the primary multivariable model. Neonatal white blood cell count was not included in the primary model because it was measured after birth and might represent an intermediate variable on the causal pathway between HCA and neonatal respiratory outcomes. Before construction of the multivariable model, multicollinearity among the selected variables was assessed. The VIF values ranged from 1.083 to 2.384, and the corresponding tolerance values ranged from 0.419 to 0.923. The VIF values for gestational age and birth weight were 2.384 and 2.327, respectively. All VIF values were <5 and all tolerance values were >0.20, indicating no substantial multicollinearity among the variables included in the primary model. Detailed multicollinearity diagnostics are presented in Table 7. After confirmation of the absence of substantial multicollinearity, the primary multivariable logistic regression model was constructed. After adjustment for gestational age, birth weight, premature rupture of membranes, antenatal corticosteroid exposure, and maternal C-reactive protein level, HCA remained independently associated with lower odds of RDS (adjusted OR = 0.654, 95% CI: 0.472–0.906, *P* = 0.011). Each 1-week increase in gestational age was associated with lower odds of RDS (adjusted OR = 0.765, 95% CI: 0.645–0.907, *P* = 0.003), and each 100-g increase in birth weight was also associated with lower odds of RDS (adjusted OR = 0.834, 95% CI: 0.740–0.941, *P* = 0.003). Premature rupture of membranes was not independently associated with RDS (adjusted OR = 0.916, 95% CI: 0.648–1.295, *P* = 0.619). Antenatal corticosteroid exposure was associated with lower odds of RDS, although the association did not reach statistical significance after multivariable adjustment (adjusted OR = 0.756, 95% CI: 0.542–1.054, *P* = 0.099). Maternal C-reactive protein level was not independently associated with RDS (adjusted OR = 1.008, 95% CI: 0.992–1.024, *P* = 0.331). The primary multivariable analysis results are presented in Table 8. In the sensitivity analysis, neonatal white blood cell count was additionally included in the primary model. The direction and magnitude of the association between HCA and RDS remained similar (adjusted OR = 0.668, 95% CI: 0.479–0.932, *P* = 0.018). Gestational age and birth weight also remained independently associated with lower odds of RDS. Neonatal white blood cell count itself was not independently associated with RDS (adjusted OR = 1.012, 95% CI: 0.979–1.046, *P* = 0.481). These findings supported the robustness of the primary analysis. The complete sensitivity analysis results are presented in Table 9. Formal interaction testing was performed by adding an interaction term between HCA status and gestational-age group to the multivariable model. The interaction between HCA and gestational-age group was not statistically significant (*β*=−0.284, SE = 0.214, Wald *χ*^2^ = 1.764, *P* for interaction=0.184). Therefore, the statistically significant association observed in the <32-week subgroup and the nonsignificant association observed in the 32–<34-week subgroup did not provide sufficient evidence that gestational age modified the association between HCA and RDS.

**Table 6:** Univariate logistic regression analysis for RDS.

Variables	β	SE	Wald *χ*^2^	*P* value	OR	95% CI
HCA	−0.549	0.161	11.657	<0.001	0.577	0.421∼0.791
Gestational age	−0.380	0.058	42.897	<0.001	0.684	0.613∼0.764
Birth weight	−0.214	0.041	27.231	<0.001	0.807	0.744∼0.875
Sex	0.082	0.158	0.270	0.603	1.085	0.797∼1.477
1-min Apgar score	−0.187	0.071	6.935	0.008	0.830	0.718∼0.958
5-min Apgar score	−0.221	0.089	6.168	0.013	0.802	0.672∼0.958
Cesarean delivery	−0.113	0.164	0.474	0.491	0.893	0.649∼1.228
PROM	−0.136	0.173	0.617	0.432	0.873	0.623∼1.223
Maternal fever	0.264	0.129	4.191	0.041	1.302	1.011∼1.677
Positive vaginal smear	0.118	0.142	0.690	0.406	1.125	0.851∼1.487
Antenatal corticosteroids	−0.298	0.148	4.052	0.044	0.742	0.555∼0.992
Antenatal magnesium sulfate	−0.071	0.151	0.221	0.638	0.932	0.693∼1.253
Antenatal antibiotics	0.105	0.159	0.435	0.510	1.111	0.815∼1.515
Maternal age	−0.021	0.015	1.960	0.161	0.979	0.951∼1.009
WBC	0.018	0.016	1.266	0.260	1.018	0.986∼1.051
NEUT	0.022	0.019	1.338	0.247	1.022	0.984∼1.061
LYMPH	−0.031	0.028	1.224	0.269	0.970	0.918∼1.026
PLT	−0.002	0.001	2.893	0.089	0.998	0.996∼1.000
RBC	−0.148	0.112	1.749	0.186	0.862	0.693∼1.073
HGB	−0.006	0.004	2.250	0.134	0.994	0.987∼1.002
SGA	0.173	0.242	0.513	0.474	1.189	0.740∼1.911

**Table 7:** Multicollinearity diagnostics for variables included in the primary RDS model.

Variables	Tolerance	VIF
HCA	0.778	1.286
Gestational age	0.419	2.384
Birth weight	0.430	2.327
Premature rupture of membranes	0.838	1.194
Antenatal corticosteroid exposure	0.923	1.083
Maternal CRP	0.821	1.218

A tolerance value >0.20 and a VIF <5 were considered to indicate the absence of substantial multicollinearity.

HCA, histological chorioamnionitis; CRP, C-reactive protein; VIF, variance inflation factor.

**Table 8:** Multivariable logistic regression analysis for RDS.

Variables	β	SE	Wald *χ*^2^	*P* value	OR	95% CI
HCA	−0.425	0.168	6.400	0.011	0.654	0.472–0.906
Gestational age, per 1-week increase	−0.268	0.089	9.074	0.003	0.765	0.645–0.907
Birth weight, per 100-g increase	−0.182	0.061	8.889	0.003	0.834	0.740–0.941
Premature rupture of membranes	−0.088	0.177	0.247	0.619	0.916	0.648–1.295
Antenatal corticosteroid exposure	−0.280	0.170	2.717	0.099	0.756	0.542–1.054
Maternal CRP	0.008	0.008	0.947	0.331	1.008	0.992–1.024

Birth weight was entered into the model per 100-g increase.

HCA, histological chorioamnionitis; CRP, C-reactive protein; OR, odds ratio; CI, confidence interval.

**Table 9:** Sensitivity analysis additionally adjusting for neonatal WBC count.

Variables	β	SE	Wald *χ*^2^	*P* value	OR	95% CI
HCA	−0.403	0.170	5.616	0.018	0.668	0.479–0.932
Gestational age, per 1-week increase	−0.259	0.087	8.861	0.003	0.772	0.650–0.916
Birth weight, per 100-g increase	−0.172	0.061	7.951	0.005	0.842	0.747–0.949
Premature rupture of membranes	−0.079	0.177	0.199	0.656	0.924	0.653–1.307
Antenatal corticosteroid exposure	−0.273	0.170	2.578	0.108	0.761	0.545–1.062
Maternal CRP	0.007	0.008	0.736	0.391	1.007	0.991–1.023
Neonatal WBC count	0.012	0.017	0.496	0.481	1.012	0.979–1.046

The sensitivity model included all variables in the primary model with additional adjustment for neonatal WBC count measured after birth.

HCA, histological chorioamnionitis; CRP, C-reactive protein; WBC, white blood cell; OR, odds ratio; CI, confidence interval.

### 3.5 Exploratory predictive performance, incremental value, and internal validation

**Figure 3:**
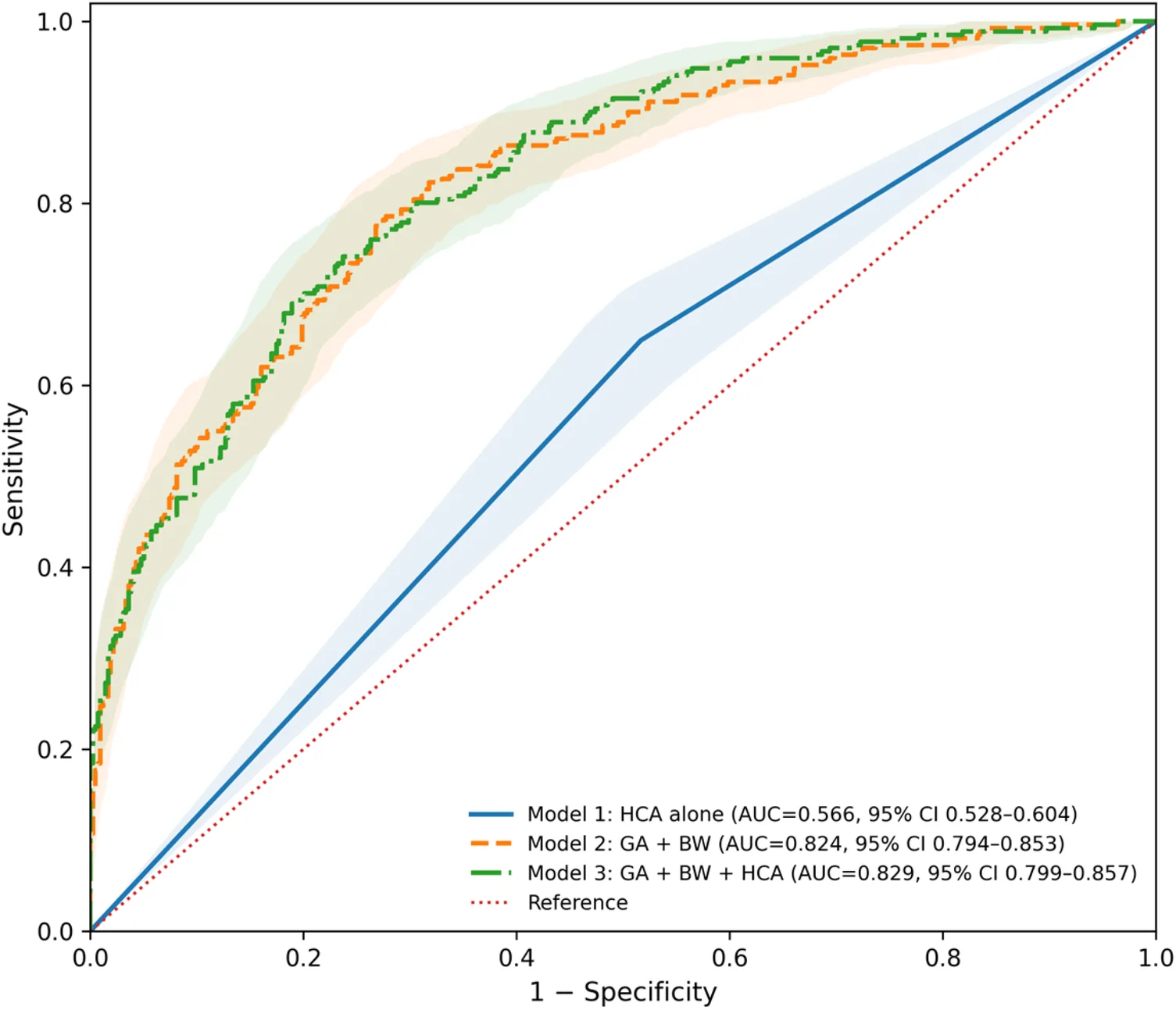
Receiver operating characteristic curves for model 1 (HCA alone), model 2 (gestational age and birth weight), and model 3 (gestational age, birth weight, and HCA). Shaded areas represent bootstrap 95% confidence bands.

**Figure 4:**
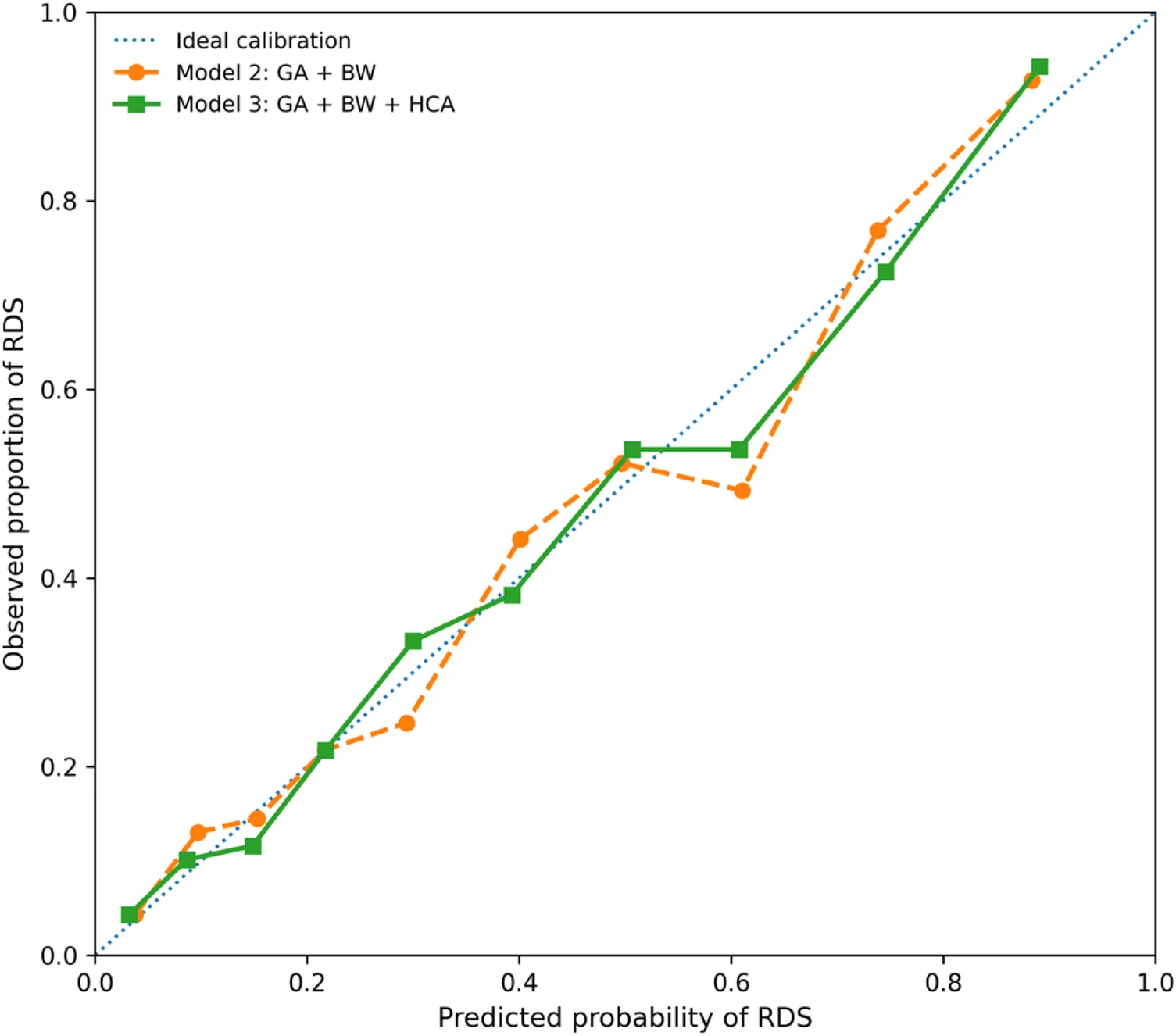
Bootstrap-corrected calibration curves for model 2 (gestational age and birth weight) and model 3 (gestational age, birth weight, and HCA). The diagonal line represents ideal calibration.

Three exploratory logistic regression models were evaluated for RDS. Model 1 included HCA alone, Model 2 included gestational age and birth weight, and Model 3 additionally incorporated HCA into Model 2.HCA alone showed poor discriminatory ability for RDS, with an AUC of 0.566 (95% CI: 0.528–0.604), a sensitivity of 64.94%, and a specificity of 48.33%. Therefore, HCA alone was not considered a clinically useful standalone predictor of RDS. Model 2, which included gestational age and birth weight, achieved an AUC of 0.824 (95% CI: 0.794–0.853), with a sensitivity of 81.18% and a specificity of 67.46%. After HCA was added, Model 3 yielded an AUC of 0.829 (95% CI: 0.799–0.857), with a sensitivity of 82.66% and a specificity of 68.66%. The absolute increase in AUC from Model 2 to Model 3 was 0.005. The DeLong test showed that the difference in AUC between the two models was not statistically significant (*Z* = 1.231, *P* = 0.218), as shown in Figure 3. Although the increase in AUC was limited, the likelihood-ratio test showed that adding HCA significantly improved the overall model fit compared with the model containing gestational age and birth weight alone (likelihood-ratio *χ*^2^ = 6.712, *P* = 0.010). The continuous net reclassification improvement was 0.118 (95% CI: 0.012–0.224, *P* = 0.030), and the integrated discrimination improvement was 0.006 (95% CI: 0.001–0.012, *P* = 0.022). These findings indicated that HCA provided a statistically detectable but quantitatively modest incremental contribution beyond gestational age and birth weight. Internal validation was performed using 1,000 bootstrap resamples. The optimism-corrected AUCs were 0.821 for Model 2 and 0.825 for Model 3, corresponding to optimism estimates of 0.003 and 0.004, respectively. For Model 3, the bootstrap-corrected calibration intercept was −0.018, the calibration slope was 0.971, and the Brier score was 0.158. The calibration curve demonstrated acceptable agreement between the predicted and observed probabilities of RDS, although slight overestimation was observed among infants with higher predicted risks, as shown in Figure 4. Detailed comparisons of model discrimination, incremental predictive value, calibration, and internal validation are presented in Table 10.

**Table 10:** Discrimination, incremental predictive value, calibration, and internal validation of the exploratory models.

Model	Predictors	Apparent AUC (95% CI)	Bootstrap-corrected AUC	Sensitivity (%)	Specificity (%)	ΔAUC vs. Model 2; *P*^a^	Likelihood-ratio test	Continuous NRI (95% CI); *P*	IDI (95% CI); P	Calibration intercept	Calibration slope	Brier score
Model 1: HCA alone	HCA	0.566 (0.528–0.604)	0.564	64.94	48.33	–	–	–	–	–	–	0.236
Model 2: Developmental model	Gestational age + birth weight	0.824 (0.794–0.853)	0.821	81.18	67.46	Reference	Reference	Reference	Reference	−0.012	0.978	0.161
Model 3: Extended model	Gestational age + birth weight + HCA	0.829 (0.799–0.857)	0.825	82.66	68.66	0.005; *P* = 0.218	*χ*^2^ = 6.712; *P* = 0.010	0.118 (0.012–0.224); *P* = 0.030	0.006 (0.001–0.012); *P* = 0.022	−0.018	0.971	0.158

Model 1 included HCA alone; Model 2 included gestational age and birth weight; Model 3 additionally included HCA.

AUC, area under the receiver operating characteristic curve; CI, confidence interval; HCA, histological chorioamnionitis; NRI, net reclassification improvement; IDI, integrated discrimination improvement.

a AUCs for Models 2 and 3 were compared using the DeLong test. Internal validation was performed using 1,000 bootstrap resamples. Predictive analyses were exploratory.

## 4 Discussion

Respiratory distress syndrome (RDS) remains a major cause of early respiratory morbidity and mortality among preterm infants. Its development is primarily related to structural immaturity of the fetal lung and insufficient synthesis and secretion of pulmonary surfactant. Gestational age and birth weight are well-established determinants of RDS because both are closely associated with the degree of pulmonary maturation (11, 12). In addition to developmental immaturity, increasing evidence suggests that intrauterine inflammatory exposure may influence fetal lung development and modify neonatal respiratory outcomes (13). Histological chorioamnionitis (HCA), a pathological manifestation of intrauterine inflammation, predominantly reflects a maternal inflammatory response but may coexist with fetal inflammatory involvement. Its association with neonatal respiratory outcomes may vary according to gestational age, inflammatory severity, duration of exposure, extension to the fetal compartment, and perinatal management (14). Therefore, a simple classification of HCA as present or absent may not fully characterize this complex relationship. In the present study, we evaluated the independent association between placental HCA and RDS after adjustment for major maternal and perinatal confounders and further explored whether the observed association differed according to gestational-age strata and the histological stage of the maternal inflammatory response.

Among preterm infants born at <34 weeks of gestation, the incidence of RDS was lower in the HCA-positive group than in the HCA-negative group. After adjustment for gestational age, birth weight, premature rupture of membranes, antenatal corticosteroid exposure, and maternal C-reactive protein level, HCA remained independently associated with lower odds of RDS (adjusted OR = 0.654, 95% CI: 0.472–0.906). This finding indicates an inverse association between placental HCA and RDS after accounting for major perinatal factors but does not establish that intrauterine inflammation has a causal protective effect. Because this was a retrospective observational study, residual confounding, differences in gestational-age distribution, selective placental examination, and unmeasured perinatal management factors may also have contributed to the observed association.

Gestational age and birth weight were both retained in the primary model because they represent related but clinically distinct dimensions of neonatal maturity and intrauterine growth. Although the two variables were correlated, their VIF values were 2.384 and 2.327, respectively, and their corresponding tolerance values were greater than 0.20. These results indicated that their simultaneous inclusion did not produce substantial multicollinearity. Neonatal white blood cell count was not included in the primary adjustment model because it was measured after birth and may represent a biological response to intrauterine inflammatory exposure. It could therefore lie on the causal pathway between HCA and neonatal respiratory outcomes, and adjustment for this potential intermediate variable might lead to overadjustment. In the sensitivity analysis additionally adjusting for neonatal white blood cell count, the direction and magnitude of the association remained similar (adjusted OR = 0.668, 95% CI: 0.479–0.932), supporting the relative robustness of the primary finding.

Previous studies have reported inconsistent associations between HCA and neonatal respiratory outcomes. Liu et al. (15) found that isolated maternal inflammatory exposure was associated with a lower incidence of RDS among extremely preterm infants, suggesting that selected prenatal inflammatory stimuli may be associated with accelerated fetal lung maturation. Hung et al. (16) reported that both isolated HCA and HCA accompanied by funisitis were independently associated with lower odds of severe RDS among very-low-birth-weight infants after adjustment for gestational age and other potential confounders. However, funisitis, which reflects a fetal inflammatory response, was also associated with a higher risk of chronic lung disease and other adverse respiratory outcomes than isolated maternal inflammation. These findings indicate that the short-term respiratory effects of intrauterine inflammation may differ from its effects on subsequent inflammatory lung injury.

In the present study, the inverse association between HCA and RDS appeared more evident among infants born at <32 weeks than among those born at 32–<34 weeks. However, formal interaction testing did not identify a statistically significant interaction between HCA and gestational-age group (*P* for interaction=0.184). A statistically significant association within one subgroup and a nonsignificant association within another subgroup do not by themselves demonstrate a true between-group difference. Therefore, the gestational-age-stratified findings should be considered exploratory and should not be interpreted as confirmed evidence that gestational age modifies the association between HCA and RDS. Larger studies with adequate statistical power and prespecified interaction analyses are required to determine whether the effect of intrauterine inflammatory exposure differs across stages of prematurity. Other studies have suggested that HCA may be associated with adverse respiratory outcomes, particularly when inflammation extends to the fetal compartment or is accompanied by funisitis (17). Kovács et al. (18) reported that HCA combined with fetal inflammatory response may be associated with an increased risk of bronchopulmonary dysplasia. These apparently conflicting findings may reflect differences in inflammatory phenotype, severity, duration, gestational timing, and fetal involvement. A limited or transient maternal inflammatory response may coincide with accelerated surfactant maturation, whereas sustained or severe inflammation extending to the fetal circulation may impair alveolarization, disrupt pulmonary vascular development, and increase the risk of chronic lung injury. Therefore, maternal and fetal inflammatory responses should not be considered biologically equivalent. Several mechanisms may explain the observed inverse association between HCA and RDS. First, prenatal inflammatory exposure may activate pathways involved in fetal lung maturation. Experimental studies have suggested that proinflammatory cytokines, including interleukin-1β and interleukin-6, may promote the expression of surfactant proteins such as SP-A and SP-B through NF-κB- and STAT3-related signaling pathways and enhance the differentiation of type II alveolar epithelial cells (19). Inflammation may also activate endogenous glucocorticoid pathways and interact with antenatal corticosteroid exposure. In the present cohort, the proportion of antenatal corticosteroid exposure was similar between the HCA-positive and HCA-negative groups, suggesting that the observed association was unlikely to be explained solely by differences in corticosteroid administration. Nevertheless, residual confounding related to the timing, completeness, number of courses, and interval between corticosteroid administration and delivery could not be excluded. Second, the association between intrauterine inflammation and respiratory outcomes may depend on the developmental stage of the fetal lung. Before 32 weeks of gestation, the fetal lung is transitioning from the canalicular to the saccular stage and may be particularly responsive to maturational stimuli. Inflammatory signaling during this period may increase surfactant synthesis and reduce the occurrence of classical surfactant-deficiency RDS. With advancing gestational age, baseline pulmonary maturity improves, and any additional maturation-related influence of inflammation may become less apparent. This interpretation is broadly consistent with previous clinical evidence showing that the respiratory consequences of intrauterine inflammation differ according to gestational age and degree of prematurity (20). However, because the formal interaction test in the present study was nonsignificant, this biological explanation remains hypothetical. Third, histological stage may reflect differences in inflammatory burden. The incidences of RDS were 26.32%, 30.61%, and 55.56% among infants with stage I, stage II, and stage III HCA, respectively. The higher incidence observed in the stage III group suggests that more extensive or prolonged inflammation may be less closely associated with pulmonary maturation and may instead coexist with inflammatory lung injury. This finding should be interpreted cautiously because only 36 infants had stage III HCA, resulting in limited statistical precision. Moreover, stage-specific adjusted associations were not estimated, and the results do not demonstrate that stage I or stage II HCA has a protective biological effect or that stage III HCA independently increases RDS risk. Nomiyama et al. (21) proposed an “inflammatory load” hypothesis, according to which increasing inflammatory severity and extension to the fetal compartment may shift the balance from short-term pulmonary maturation toward chronic respiratory injury. This hypothesis may partly explain why mild or isolated maternal inflammation has sometimes been associated with a lower incidence of early RDS, whereas fetal inflammatory response has been linked to bronchopulmonary dysplasia and other adverse outcomes. Future studies should therefore distinguish maternal inflammatory response from fetal inflammatory response and incorporate funisitis, chorionic plate vasculitis, cord-blood cytokines, and other inflammatory biomarkers into stage-specific analyses. The histological stages evaluated in the present study primarily represented the maternal inflammatory response. Fetal inflammatory response, commonly manifested as funisitis or chorionic plate vasculitis, was not systematically recorded in the original pathological reports and could not be analyzed separately. Because fetal inflammatory involvement may have effects on neonatal respiratory outcomes that differ substantially from those of isolated maternal inflammation, the present findings should not be generalized to infants with confirmed fetal inflammatory response. Selective placental examination according to clinical indications may also have resulted in overrepresentation of pregnancies complicated by premature rupture of membranes, maternal fever, suspected infection, or other high-risk conditions. Accordingly, the observed inverse association should be interpreted as an epidemiological association within the selected cohort rather than as evidence that HCA directly protects against RDS.

Fetal growth status may also contribute to neonatal respiratory outcomes. Previous studies have reported inconsistent associations between small for gestational age (SGA) status and RDS, possibly because SGA represents a heterogeneous condition arising from different maternal, placental, and fetal causes. In the present study, SGA was not significantly associated with RDS in the univariate analysis. Because SGA was defined using gestational age-, sex-, and birth weight-specific percentiles, it was not entered simultaneously with gestational age and birth weight in the multivariable model to avoid redundant adjustment and potential collinearity. However, the absence of a statistically significant association should not be interpreted as evidence that fetal growth status has no influence on RDS risk. Differences in the underlying causes and severity of fetal growth restriction, as well as variation in the growth reference used to define SGA, may partly explain the inconsistent findings across studies.

The predictive analyses further support a cautious interpretation of the clinical value of HCA. HCA alone showed poor discriminatory ability for RDS, with an AUC of 0.566, indicating that it should not be regarded as a clinically useful standalone predictor. By contrast, the developmental model incorporating gestational age and birth weight achieved an AUC of 0.824, suggesting that pulmonary maturity and fetal growth accounted for most of the model's discriminatory performance. This finding is consistent with the concept proposed by Jang et al. (22) that a single inflammatory exposure variable is insufficient to characterize RDS risk and should be interpreted together with developmental and perinatal factors.

After HCA was added to gestational age and birth weight, the AUC increased only slightly from 0.824 to 0.829, and the difference was not statistically significant according to the DeLong test (*P* = 0.218). Although the likelihood-ratio test, continuous net reclassification improvement, and integrated discrimination improvement showed statistically significant changes, their absolute magnitudes were small. These results indicate that HCA provided a statistically detectable but quantitatively modest incremental contribution beyond gestational age and birth weight and did not meaningfully improve overall model discrimination. Statistical improvements in model fit or risk reclassification should therefore not be interpreted automatically as clinically important improvements in prediction. Bootstrap internal validation showed limited optimism, with optimism-corrected AUCs of 0.821 for the developmental model and 0.825 for the extended model, and the extended model demonstrated acceptable calibration. Nevertheless, these findings were derived from a single retrospective cohort and require independent external validation. In addition, placental histopathological results are generally unavailable during the initial diagnosis and respiratory management of RDS. Accordingly, the extended model should be regarded as an exploratory analytical model rather than a real-time clinical prediction tool.

The adjusted analyses did not identify statistically significant associations between HCA and early-onset sepsis, bronchopulmonary dysplasia, retinopathy of prematurity, intraventricular hemorrhage, or necrotizing enterocolitis. Although the unadjusted incidence of early-onset sepsis was higher in the HCA-positive group, this association was attenuated after adjustment for relevant perinatal factors. Therefore, the lower incidence of RDS among HCA-positive infants should not be interpreted as evidence of uniformly favorable neonatal outcomes. HCA reflects intrauterine inflammatory exposure and may coexist with infection-related and inflammatory complications. The absence of an adjusted association between HCA and bronchopulmonary dysplasia differs from previous reports suggesting that intrauterine inflammation, particularly when accompanied by fetal inflammatory response, may increase the risk of bronchopulmonary dysplasia (23). This discrepancy may be related to differences in gestational-age distribution, inflammatory phenotype, fetal involvement, duration of mechanical ventilation, cumulative oxygen exposure, surfactant treatment, postnatal infection, and other factors involved in the multifactorial pathogenesis of bronchopulmonary dysplasia. In addition, the present cohort included infants born at up to 34 weeks of gestation, whereas bronchopulmonary dysplasia occurs predominantly among very and extremely preterm infants. The relatively broader gestational-age range may therefore have reduced the ability to detect an association between HCA and bronchopulmonary dysplasia.

A major limitation to the clinical application of HCA is the delay between delivery and the availability of placental histopathological results. The diagnosis and initial management of neonatal RDS should be based on gestational age, respiratory signs, oxygen requirement, blood gas findings, chest imaging, and the infant's response to respiratory support. Clinical assessment and treatment should not be delayed while awaiting placental pathological examination. Accordingly, HCA should not be regarded as a real-time diagnostic marker for RDS or as a substitute for established neonatal assessment and management criteria. After placental pathological results become available, HCA status may provide supplementary information regarding intrauterine inflammatory exposure and the possible etiological background of the neonatal respiratory phenotype. In clinical interpretation, HCA should be considered together with gestational age, birth weight, fetal growth status, antenatal corticosteroid exposure, and the postnatal respiratory course. An HCA-positive result may contribute to etiological interpretation, particularly among highly immature infants, but it should not independently justify a reduction or withdrawal of oxygen therapy, noninvasive ventilation, mechanical ventilation, or other respiratory support. Placental evidence of inflammation may also prompt closer evaluation for infection-related or inflammatory complications, but any subsequent management decision should remain based on the infant's clinical condition and established diagnostic criteria. The present study did not evaluate whether the availability of placental pathological information altered clinical management or improved outcomes such as mortality, bronchopulmonary dysplasia, duration of mechanical ventilation, oxygen dependence, or length of hospitalization. Therefore, the findings support HCA only as supplementary postnatal information for etiological interpretation and retrospective risk characterization. They do not demonstrate that pathology-informed management directly improves neonatal outcomes or that HCA should be incorporated into real-time respiratory decision-making.

Several limitations should be acknowledged. First, this was a single-center retrospective cohort study based on existing clinical records; therefore, selection bias, information bias, and residual confounding cannot be completely excluded. Placental histopathological examination was performed selectively according to clinical indications rather than routinely in all preterm deliveries. Pregnancies complicated by premature rupture of membranes, maternal fever, suspected intrauterine infection, abnormal amniotic fluid, or other high-risk conditions were more likely to undergo placental examination. Consequently, the included cohort may not fully represent all preterm infants born before 34 weeks of gestation, and the generalizability of the findings may be limited. Second, although the maternal inflammatory response was classified according to the Amsterdam Placental Workshop Group criteria, fetal inflammatory response, including funisitis and chorionic plate vasculitis, was not systematically recorded in the original pathological reports and could not be analyzed separately. Maternal and fetal inflammatory responses may have different effects on pulmonary maturation and inflammatory lung injury. The absence of fetal inflammatory response data may therefore have introduced residual heterogeneity and limited interpretation of the associations according to inflammatory phenotype and histological stage. Third, only 36 infants had stage III HCA, resulting in limited statistical precision for stage-specific comparisons. In addition, the gestational-age-stratified and histological-stage analyses were exploratory. The nonsignificant interaction test did not confirm that gestational age modified the association between HCA and RDS, and the observed differences across histological stages should not be interpreted as evidence of a dose-response relationship. Fourth, several potentially relevant perinatal and postnatal variables were not fully available or incorporated into the models, including the timing, completeness, and interval of antenatal corticosteroid therapy, the duration of membrane rupture, surfactant administration, the type and duration of respiratory support, cumulative oxygen exposure, and postnatal infection. Residual confounding by these unmeasured or incompletely measured factors therefore remains possible. Fifth, the study focused primarily on the occurrence of early RDS and did not include long-term follow-up of pulmonary function, recurrent respiratory morbidity, oxygen dependence, or other chronic respiratory outcomes. The long-term respiratory implications of HCA exposure could therefore not be evaluated. Sixth, although internal validation using 1,000 bootstrap resamples showed limited optimism and acceptable calibration, the exploratory models were developed and validated within the same single-center cohort. Independent external validation was not performed, and the transportability of the discrimination and calibration results to other populations remains uncertain. Moreover, the incremental predictive contribution of HCA beyond gestational age and birth weight was quantitatively modest and requires further clinical evaluation. Finally, placental histopathological results were generally unavailable during the initial diagnosis and respiratory management of RDS. HCA therefore cannot serve as a real-time clinical predictor, and the present study did not assess whether pathology-informed management altered treatment decisions or improved neonatal outcomes. Prospective multicenter studies incorporating standardized placental examination, separate assessment of maternal and fetal inflammatory responses, detailed perinatal and respiratory treatment variables, external model validation, and long-term follow-up are required to confirm these findings.

In summary, placental HCA was independently associated with lower odds of RDS among preterm infants after adjustment for major maternal and perinatal confounders. However, this inverse association should not be interpreted as evidence of a causal protective effect. Although the association appeared more pronounced among infants born before 32 weeks of gestation and varied across histological stages, the nonsignificant interaction test and the small number of infants with stage III HCA indicate that these subgroup findings remain exploratory. HCA alone showed poor discriminatory ability for RDS. Adding HCA to a model containing gestational age and birth weight produced statistically detectable improvements in model fit and risk reclassification but only a small and nonsignificant increase in AUC. Therefore, the incremental predictive value of HCA should be considered modest, and HCA should not be regarded as a useful standalone or real-time clinical predictor. Once placental pathological results become available, HCA may instead provide supplementary postnatal information for etiological interpretation and retrospective risk characterization when considered together with gestational age, birth weight, fetal growth status, and the infant's clinical respiratory course.

## 5 Conclusions

In conclusion, placental histological chorioamnionitis was independently associated with lower odds of respiratory distress syndrome in preterm infants after adjustment for major perinatal confounders. HCA alone showed limited discriminatory ability and should not be considered a real-time clinical predictor. Its potential value lies in supplementary etiological interpretation and postnatal risk stratification when integrated with gestational age, birth weight, and other clinical factors.

## Data Availability

The datasets generated and/or analyzed during the current study are available from the corresponding author upon reasonable request.
